# Proton Therapy for Skull-Base Chordomas and Chondrosarcomas: Initial Results From the Beaumont Proton Therapy Center

**DOI:** 10.7759/cureus.15278

**Published:** 2021-05-27

**Authors:** Jacob S Parzen, Xiaoqiang Li, Weili Zheng, Xuanfeng Ding, Peyman Kabolizadeh

**Affiliations:** 1 Department of Radiation Oncology, Beaumont Proton Therapy Center, Oakland University William Beaumont School of Medicine, Royal Oak, USA

**Keywords:** proton beam therapy, skull-base chordomas, skull-base chondrosarcomas, pencil beam scanning, simultaneous integrated boost

## Abstract

Background:Skull-base chordomas and chondrosarcomas are rare tumors that arise directly adjacent to important critical structures. Appropriate management consists of maximal safe resection followed by postoperative dose-escalated radiation therapy. Proton beam therapy is often employed in this context to maximize the sparing of organs at risk, such as the brainstem and optic apparatus.

Methods: This is a single-institutional experience treating skull-base chordomas and chondrosarcomas with postoperative pencil beam scanning proton therapy. We employed a simultaneous integrated boost to the gross tumor volume (GTV) for increased conformality. Demographic, clinicopathologic, toxicity, and dosimetry information were collected. Toxicity was assessed according to Common Terminology Criteria for Adverse Events (CTCAE), v. 4.0.

Results: Between 2017 and 2020, 13 patients were treated with postoperative proton therapy. There were 10 patients with chordoma (77%) and three with chondrosarcoma (23%). A gross total resection was achieved in six (60%) patients with chordoma and one patient with chondrosarcoma (33%). Nine patients (69%) received postoperative therapy, whereas four (31%) received treatment at recurrence/progression following re-excision. The median dose to the GTV was 72.4 cobalt-Gray equivalents (range, 70.0 to 75.8). The mean GTV was 3.4 cc (range, 0.2-38.7). There were no grade 3 or greater toxicities. One patient developed grade 2 temporal lobe necrosis. At 10.7 months' median follow-up (range, 2.1-30.6), the rates of local control and overall survival were 100%.

Conclusions: Proton beam therapy with pencil beam scanning and simultaneous integrated boost to the GTV affords excellent early local control with the suggestion of low morbidity. This method deserves consideration as an optimal method for limiting dose to adjacent organs at risk and delivering clinically effective doses to the treatment volume.

## Introduction

Skull-base chordomas and chondrosarcomas are rare tumors that are therapeutically challenging due to their proximity to the brainstem, optic apparatus, and temporal lobes of the brain. Owing to low rates of metastases, patients typically succumb to local progression [1,2]. As a result, local therapies are of paramount importance. In general, treatment consists of maximal safe surgical resection followed by postoperative radiotherapy. Unfortunately, gross total resection is not always possible [3]. This may be of particular detriment to patients with chordomas, for whom gross total resection is of relatively greater importance. Historically, photon-based therapies have been utilized, but the inability to achieve therapeutic dosing while respecting normal tissue constraints resulted in inferior clinical outcomes [4,5]. To achieve dose >70 cobalt-gray equivalents (CGE), proton beam therapy (PBT) is now often utilized at specialized centers offering this approach [6] and results in local control above 90% for chondrosarcomas and 60-80% for chordomas [7-9].

To date, much of the published literature has evaluated the use of passive scatter protons [10]. Notable experiences from Massachusetts General Hospital (MGH) and Loma Linda University have outlined initial treatment to a low-risk volume followed by a sequential cone-down to a high-risk volume [7,8]. There have been limited reports on active scanning or intensity-modulated proton therapy (IMPT) [11,12]. Passive scattering requires field-specific and patient-specific hardware and is more prone to neutron contamination. Active scanning techniques, such as spot or pencil beam, allow for better conformality of the proximal edge of the treatment volume and are better equipped to conform to target volumes constrained by critical structures [13]. This is achieved by accumulating mono-energetic pencil-proton beamlets to dynamically “paint” the target volume. As of 2020, virtually all new proton facilities employ active scanning. Since 2017, we have exclusively treated patients with pencil beam scanning (PBS) technique and employed simultaneous integrated boost (SIB) for increased conformality and better sparing of critical structures when compared to a sequential boost to the high-risk volume. This approach avoids overlap and accumulation of dose outside the target volume which presents a limitation to treatment with a sequential cone-down boost [12]. As both chordomas and chondrosarcomas have an estimated α/β ratio of 2 [14], the use of SIB also results in a higher biological equivalent dose (BED) compared to 1.8 Gy fractions, which may also result in improved tumor control based on the linear-quadratic model. Herein, we present preliminary outcomes with this approach, with a focus on acute and late toxicity, dosimetric parameters, local control, and overall survival.

## Materials and methods

Patients

Following institutional review board approval, we queried our proton database for all patients undergoing postoperative PBT for skull-base chordomas or chondrosarcomas. In general, patients were also enrolled on REG001-09, a prospective, multi-institutional registry of proton patients. All patients had histologically confirmed disease and were treated with curative intent. Upon magnetic resonance imaging (MRI) at the time of treatment planning, all patients had some degree of residual disease. No patient presented with Ollier’s disease or Maffucci syndrome. The patient's tumor characteristics, radiation treatment details, toxicity, and dosimetric information were all collected.

Proton beam radiotherapy

Patients underwent three-dimensional CT simulation with a helical CT scanner (Philips Brilliance Big Bore, Philips Healthcare System, Cleveland, OH). Intravenous iodinated contrast was utilized. Diagnostic and departmental MRI with T1, T2, and T1 fat-suppressed sequences was completed in the treatment position for optimal image fusion and target/organ-at-risk (OAR) delineation. Gross tumor volume (GTV) was defined as macroscopic tumor as identified on CT and MRI scans. Clinical target volume 1 (CTV1) consisted of the preoperative disease extent with coverage of all intermediate-risk subclinical extent of disease per physician discretion which included the surgical bed plus any area at risk. CTV2 consisted of all high-risk subclinical extent of disease including the surgical bed. Both CTV1 and CTV2 included the GTV. All target volume delineation was completed by a fellowship-trained proton radiation oncologist. Strict adherence to the dose constraints in Table 1 was mandated and would not be sacrificed to enhance target coverage. The brainstem center was defined as the brainstem with an isotropic 3 mm reduction. All patients were treated with IMPT based on the PBS technique via 3D imaging guidance with cone-beam computed tomography (CBCT). Plans were generated using a five-beam combination of left anterior oblique, left posterior oblique, posteroanterior, right anterior oblique, right posterior oblique and/or vertex beams. Planning was completed with RayStation version 6 (RaySearch Laboratories AB, Stockholm, Sweden) and treatment delivered with the IBA ProteusONE beam model (Ion Beam Application SA, Belgium). Monte Carlo algorithms were used for particle dose calculations. Robust optimization parameters were set to ±3.5% range and ±2 mm in x, y, z directions for setup uncertainties resulting in 21 (21 for optimization, 26 for robust analysis) perturbed total dose scenarios. Robust optimization was conducted with RayStation version 6. A robust evaluation was completed with in-house scripting. The maximum dose of 0.03 cc of the volume was extracted for all OARs. The mean and standard deviations of the maximum doses for all perturbed dose scenarios for all patients were calculated based on recalculating the doses with ±3.5% range and ±2 mm setup shifts including all diagonal directions (total 26 scenarios). All constraints were also evaluated via RayStation dose-volume histogram (DVH) analysis. This two-step verification with both robust optimization and robust evaluation ensured safety while using IMPT. The relative biologic effectiveness (RBE) was set at 1.1 per institutional standard. CGE was proton dose in Gy multiplied by RBE. All patients were intended to be treated with proton therapy alone, though some received a limited number of fractions with backup photon therapy as needed. Toxicity was graded using the National Cancer Institute Common Terminology Criteria for Adverse Events (CTCAE) version 4.0.

**Table 1:** Institutional dose constraints. Abbreviations: cc, cubic centimeter; CGE, cobalt-gray equivalent.

Organ at risk	Dose constraint
Brainstem surface	Maximum dose, 0.03 cc: 65 CGE
Brainstem center	Maximum dose, 0.03 cc: 50 CGE
Optic chiasm	Maximum dose, 0.03 cc: 61 CGE
Left optic nerve	Maximum dose, 0.03 cc: 61 CGE
Right optic nerve	Maximum dose, 0.03 cc: 61 CGE

## Results

Patient characteristics

In total, 13 patients were identified, having received treatment between December 2017 and May 2020. Of the three patients with chondrosarcoma, one had grade 2 disease, and two had grade 1 disease. In addition to the presenting symptomatology in Table 2, there was one instance of each of the following: facial pain, nasal congestion, rhinorrhea, photophobia, dizziness, and gait instability.

**Table 2:** Patient characteristics. Abbreviations: RT, radiation therapy.

Patient Characteristic		No. (%) or Median (Range)
Age, years		55 (23-74)
Sex		
	Women	5 (38%)
	Men	8 (62%)
Histology		
	Chordoma	10 (77%)
	Chondrosarcoma	3 (23%)
Surgery		
	Gross total resection	7 (54%)
	Subtotal resection	6 (46%)
Indication for RT		
	Postoperative	9 (69%)
	Progression/recurrence	4 (31%)
Brainstem involvement		
	Yes	8 (62%)
	No	5 (38%)
Optic pathway involvement		
	Yes	4 (31%)
	No	9 (69%)
Cranial nerve deficit		8 (62%)
	VI	6 (46%)
Endocrine disorder		3 (23%)
Comorbidities		
	Smoking	9 (69%)
	Hyperlipidemia	4 (31%)
	Hypertension	4 (31%)
	Cardiac disease	3 (23%)
Presenting symptoms		
	Headaches	7 (54%)
	Diplopia	7 (54%)
	Change visual acuity	4 (31%)
	Facial weakness	2 (15%)
	Ptosis	2 (15%)
	Otalgia	2 (15%)

Radiation and dosimetry

**Figure 1:**
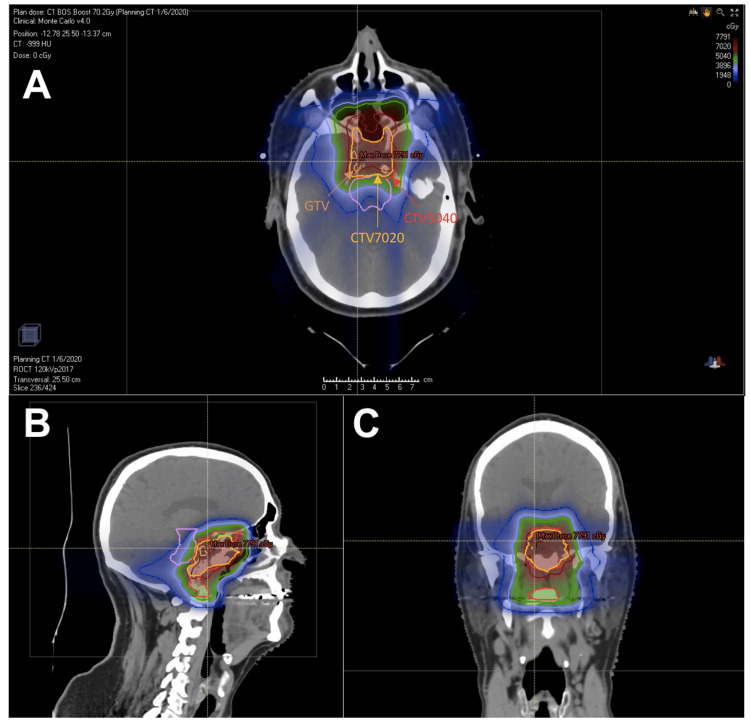
Representative (A) axial, (B) sagittal, and (C) coronal images of a patient treated with simultaneous integrated boost to 75.8 cobalt-gray equivalents to the gross tumor volume. Pertinent target volumes are denoted on the axial image. Abbreviations: CTV, clinical target volume; GTV, gross tumor volume.

**Figure 2:**
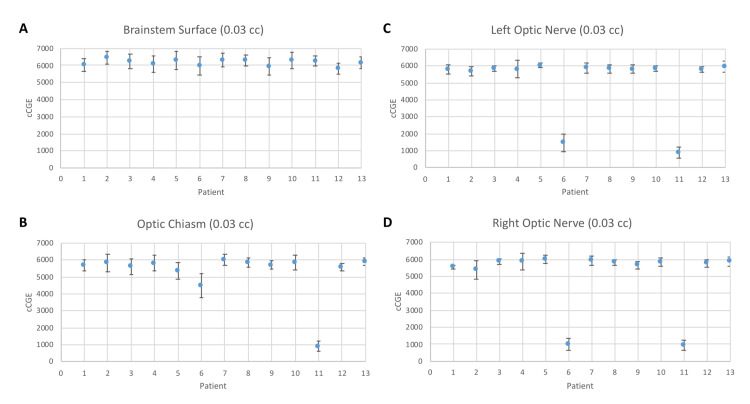
Plot of average maximum doses (0.03 cc) to the (A) brainstem surface, (B) optic chiasm, (C) left optic nerve, and (D) right optic nerve for each of the 13 patients with robustness evaluation and error bars representing standard deviations for the 26 dose scenarios. Abbreviation: cc, cubic centimeter; cCGE, centi-cobalt-gray equivalent.

All patients were treated with PBS technique and Table 3 depicts treatment characteristics and dosimetry. All patients received 50.4 CGE to the CTV1. The median dose to the CTV2 was 70.2 CGE (range, 50.4-70.2). The median prescribed dose to the GTV was 72.4 CGE (range, 70.0-75.8 CGE). Eleven out of 13 (85%) patients received an SIB to the GTV. In all cases, strict adherence to the dose constraints in Table 1 was met. Figures 1A-1C, respectively, depict axial, sagittal, and coronal representations of a representative plan in which SIB was utilized to achieve 75.8 CGE to the GTV. Figures 2A-2D present the robustness analysis of maximum dose (0.03 cc) to each of the brainstem, optic chiasm, left optic nerve, and right optic nerve. Three patients received less than or equal to three fractions with photon-based therapy due to proton machine maintenance.

**Table 3:** Tumor and treatment characteristics. Abbreviations: cc, cubic centimeter; CGE, cobalt-gray equivalent; CTV, clinical target volume; GTV, gross tumor volume.

Patient Characteristic	No. or Median (Range)
Involved site	
Cavernous sinus	5
Cervical spine	2
Clivus	13
Ethmoid	3
Petrous bone	1
Sphenoid bone	11
Suprasellar	8
Nasal cavity/nasopharynx	2
Fractions	39 (35-39)
Fraction size, primary, CGE	2 (1.8-2)
Fraction size, secondary, CGE	1.8
Target dose	
CTV1, CGE	50.4
CTV2, CGE	70.2 (50.4-70.2)
GTV, CGE	72.4 (70.0-75.8)
Target volume	
CTV2 volume, cc	26.6 (9.7-138.0)
GTV volume, cc	3.4 (0.2-38.7)
Organs-at-risk (nominal evaluation)	
Brainstem surface, 0.03 cc CGE	61.3 (57.6-64.3)
Brainstem center, 0.03 cc, CGE	41.9 (20.3-48.6)
Optic chiasm, 0.03 cc, CGE	56.8 (8.2-59.4)
Left optic nerve, 0.03 cc, CGE	57.6 (8.5-59.9)
Right optic nerve, 0.03 cc, CGE	57.3 (9.3-59.2)
Left temporal lobe, 0.03 cc CGE	71.6 (70.1-73.6)
Right temporal lobe, 0.03 cc, CGE	71.9 (32.9-78.0)
Organs-at-risk analysis (robustness evaluation)	
Brainstem surface, 0.03 cc CGE	62.3 (58.3-64.5)
Optic chiasm, 0.03 cc CGE	57.0 (8.9-60.0)
Left optic nerve, 0.03 cc CGE	58.1 (8.7-60.3)
Right optic nerve, 0.03 cc CGE	58.2 (9.3-60.0)

Clinical outcomes/toxicity

With median follow-up of 10.7 months (range, 2.1-30.6), there were no incidences of local or distant failure, and all patients were alive. Three out of 13 patients had at least 24 months of follow-up. There were no episodes of grade 3 or greater toxicity. There were two episodes of grade 1 headache (2/13, 15%), one episode of grade 1 xerostomia (1/13, 8%), one episode of grade 1 trismus (1/13, 8%), two episodes of grade 1 nausea (2/13, 15%), one episode of grade 1 mucositis (1/13, 8%), and two episodes of grades 1-2 fatigue (2/13, 15%). One patient developed grade 2 radiation necrosis of the bilateral temporal lobes, which was accompanied by grade 2 fatigue, grade 1 gait disturbance, and grade 2 cognitive disturbance. At last follow-up, the radiation necrosis had improved with supportive measures. This patient had volume receiving 70 CGE (D70) = 0.1 cc for the bilateral temporal lobes. For all patients, the median D70 to the left temporal lobe was 0.15 cc (range, 0.0-1.1) and the median D70 to the right temporal lobe was 0.15 cc (range, 0.0-1.6). One additional patient experienced grade 2 mucositis of the soft palate, for which he was re-planned with subsequent resolution of his condition.

## Discussion

Herein, we present our approach using a SIB via PBS to the GTV in patients with skull-base chordomas and chondrosarcomas. With no local failures at median 10.7 months follow-up, this treatment approach has yielded promising early clinical outcomes with no episodes of grade 3 or greater toxicity.

Although histologically distinct, skull-base chondrosarcomas and chordomas share the same management paradigm. In the largest series of proton therapy for skull-base chordomas and chondrosarcomas to date, MGH accumulated 519 patients who were treated from 66 to 83 CGE with a combined photon/proton approach [7]. With a median follow-up of 41 months, local recurrence-free survival was 98% for chondrosarcomas, 81% for male chordomas, and 65% for female chordomas. Importantly, 95% of failures were local. They employed 1.8 Gy per fraction and incorporated an initial planning volume followed sequentially by one or two cone-down boosts to the intermediate/high-risk regions. This approach has become the widespread standard amongst many institutions offering this specialized treatment. The Loma Linda experience is another notable report [8]. They accumulated 58 patients, treated to a median target dose of 70.7 CGE in 1.8 CGE fractions, with a 94% three-year local control rate for chondrosarcomas and a 67% rate for chordomas at a median of 33 months follow-up. More recently, the Paul Scherrer Institute has published the most extensively on treatment with a spot-scanning technique [15]. With chordoma patients receiving a mean dose of 73.5 CGE and chondrosarcoma patients receiving a mean dose of 68.4 CGE, they reported five-year LC rates of 81% and 94%, respectively, at median follow-up of 38 months. Long-term follow-up has confirmed excellent LC for chondrosarcomas at approximately 90% [11]. Of note, IMPT was utilized in 20 of the 64 patients in their initial report.

The majority of patients on the current study received an SIB. Investigators at MD Anderson Cancer Center have similarly described the use of SIB using spot either single-field optimized scanning beam plans or multifield-optimized IMPT [12]. At median follow-up of 27 months, they reported one local recurrence and one distant failure amongst the 15 patients included in the analysis. Similar to our report, there were no episodes of grade 3 or greater toxicity. In contrast, investigators at Paul Scherrer Institute only used IMPT for sequential boosts. In our experience, the use of SIB simplifies the process of planning and quality assurance through optimization of a single plan with dose painting. The use of 2 Gy per fraction to the GTV using the SIB PBS technique also results in a higher BED when compared to institutions where 1.8 Gy per fraction is the norm, due to the low α/β ratio of 2 for chondrosarcomas and chordomas. The shortened treatment course also theoretically inhibits repopulation of the irradiated tumor cells. We have no local failures at median 10.7 months follow-up, but longer follow-up will be necessary for further evaluation of comparative efficacy. It should be noted that previous reports of extreme hypofractionated approaches, including single-fraction and fractionated stereotactic radiosurgery (SRS), have not yielded acceptable clinical outcomes in chordomas. For instance, the University of Pittsburgh reported a low 53% five-year tumor control rate for chordomas after a single stereotactic radiosurgery procedure [16]. However, most of the patients in that series had previously undergone multiple resections and fractionated radiation therapy, suggesting a different patient population than the current series. Still, other series have similarly published inferior results for chordomas, though the treatment of chondrosarcomas with SRS may be more reasonable [17,18].

Although our follow-up is somewhat limited, a recent series on chordomas from Florida demonstrated a 14% rate of local recurrence at two years [19], suggesting that early local failures do occur and lending significant promise to our outcomes thus far. Conversely, a report from the same institution on skull-base chondrosarcomas demonstrated 100% local control at two years, with 89% local control by four years with a median follow-up of 3.7 years [20]. These differences likely reflect the more aggressive local nature of chordomas compared to chondrosarcomas. As 11 of our 13 patients had chordomas, our 100% local control rate is very promising, but a longer follow-up is needed. We acknowledge that the median GTV at PBT for our patients was 3.7 cc, which smaller than other previous series. At Indiana University, the median GTV was 8.1 cc, and GTV at the time of radiation therapy as a continuous variable was associated with improved local control [21].

Our cohort has had a very modest toxicity profile. A single patient experienced grade 2 temporal lobe necrosis which has improved with supportive care. The temporal lobe is an important OAR that typically lies in close proximity to the target volume bilaterally. Though no prospectively validated constraints exist, there is suggestion that V70 > 1.7 cc correlates with increased risk for necrosis [22]. The patient in our series met this constraint, with V70 = 0.1 cc, so there were likely other factors that predisposed them to this toxicity. The MGH experience reported five-year brainstem toxicity of 8%, five-year temporal lobe injury of 8%, and 4.4% rate of optic neuropathy [7]. Loma Linda reported a 7% rate of grade 3 or 4 toxicity, including one episode of asymptomatic temporal necrosis [8]. In the previous report of treatment with spot-scanning technique at Paul Scherrer Institute, five-year freedom from high-grade toxicity was 94% [15]. Importantly, we report no clinical toxicities to the optic apparatus or brainstem, though follow-up is limited.

We acknowledge our small sample size, a result of the rarity of these disease processes. In addition, follow-up is very limited compared to the pre-eminent series, and this may be clinically meaningful as late failures are a concern, particularly for chordomas. In addition, pathology reports did not include pathological subtyping for chordomas. Hence, we are unable to capture the rate of poorly differentiated chordomas, which are prone to early relapses. At the same time, we present one of the only series to date on PBS for skull-base chordomas and chondrosarcomas. We are one of the first to publish on the use of SIB to the GTV to spare critical structures by avoiding the accumulation of low-dose regions which may affect cumulative dose to OAR. In addition, clinical and toxicity follow-up was performed by our institution, with toxicity prospectively documented by a physician.

## Conclusions

In conclusion, pencil beam proton therapy with SIB is a promising technique to safely deliver dose-escalated treatment in skull-base chordomas and chondrosarcomas. Clinical outcomes and toxicity are very promising with early follow-up and our method merits additional consideration at centers offering this specialized treatment.
